# Gene Mutation Analysis in EGFR Wild Type NSCLC Responsive to Erlotinib: Are There Features to Guide Patient Selection?

**DOI:** 10.3390/ijms16010747

**Published:** 2014-12-31

**Authors:** Paola Ulivi, Angelo Delmonte, Elisa Chiadini, Daniele Calistri, Maximilian Papi, Marita Mariotti, Alberto Verlicchi, Angela Ragazzini, Laura Capelli, Alessandro Gamboni, Maurizio Puccetti, Alessandra Dubini, Marco Angelo Burgio, Claudia Casanova, Lucio Crinò, Dino Amadori, Claudio Dazzi

**Affiliations:** 1 Biosciences Laboratory, Istituto Scientifico Romagnolo per lo Studio e la Cura dei Tumori (IRST) IRCCS, Meldola 47014, Italy; E-Mails: elisa.chiadini@irst.emr.it (E.C.); daniele.calistri@irst.emr.it (D.C.); laura.capelli@irst.emr.it (L.C.); 2 Department of Medical Oncology, Istituto Scientifico Romagnolo per lo Studio e la Cura dei Tumori (IRST) IRCCS, Meldola 47014, Italy; E-Mails: angelo.delmonte@irst.emr.it (A.D.); marita.mariotti@irst.emr.it (M.M.); marco.burgio@irst.emr.it (M.A.B); dino.amadori@irst.emr.it (D.A.); 3 Department of Oncology, Per gli Infermi Hospital, Rimini 47900, Italy; E-Mail: maximilianpapi@libero.it; 4 Department of Medical Oncology, Santa Maria delle Croci Hospital, Ravenna 48121, Italy; E-Mails: alberto28981@gmail.com (A.V.); c.casanova@ausl.ra.it (C.C.); c.dazzi@ausl.ra.it (C.D.); 5 Unit of Biostatistics and Clinical Trials, IRST IRCCS, Meldola 47014, Italy; E-Mail: angela.ragazzini@irst.emr.it; 6 Oncology Unit, Degli Infermi Hospital, Faenza 48018, Italy; E-Mail: a.gamboni@ausl.ra.it; 7 Pathology Unit, Santa Maria delle Croci Hospital, Ravenna 48121, Italy; E-Mail: m.puccetti@ausl.ra.it; 8 Pathology Unit, Morgagni-Pierantoni Hospital, Forlì 47121, Italy; E-Mail: a.dubini@ausl.fo.it; 9 Division of Medical Oncology, Santa Maria della Misericordia Hospital, Perugia 06156, Italy; E-Mail: lucio.crino@ospedale.perugia.it

**Keywords:** NSCLC, erlotinib, *EGFR* wt, *p53*, *KRAS*

## Abstract

Tyrosine kinase inhibitors (TKIs) are very efficacious in non-small-cell lung cancer (NSCLC) patients harboring activating *Epidermal Growth Factor Receptor* (*EGFR*) mutations. However, about 10% of *EGFR* wild type (wt) patients respond to TKI, with unknown molecular mechanisms of sensitivity. We considered a case series of 34 *EGFR* wt NSCLC patients responsive to erlotinib after at least one line of therapy. Responsive patients were matched with an equal number of non-responsive *EGFR* wt patients. A panel of 26 genes, for a total of 214 somatic mutations, was analyzed by MassARRAY^®^ System (Sequenom, San Diego, CA, USA). A 15% *KRAS* mutation was observed in both groups, with a prevalence of G12C in non-responders (80% *vs.* 40% in responders). *NOTCH1*, *p53* and *EGFR*-resistance-related mutations were found more frequently in non-responders, whereas *EGFR*-sensitizing mutations and alterations in genes involved in proliferation pathways were more frequent in responders. In conclusion, our findings indicate that *p53*, *NOTCH1* and exon 20 *EGFR* mutations seem to be related to TKI resistance. *KRAS* mutations do not appear to influence the TKI response, although G12C mutation is more frequent in non-responders. Finally, the use of highly sensitive methodologies could lead to the identification of under-represented *EGFR* mutations potentially associated with TKI sensitivity.

## 1. Introduction

Treatment with tyrosine kinase inhibitors (TKIs) has shown promising results in non-small-cell lung cancer (NSCLC) patients harboring activating epidermal growth factor receptor (*EGFR*) mutations. In view of the results reported by the IPASS study [1], gefitinib (IRESSA, AstraZeneca Pharmaceuticals, Wilmington, DE, USA) was the first TKI approved by the European Medicines Agency (EMEA) for first-line therapy in adults with locally advanced or metastatic *EGFR* mutated NSCLC. Moreover, the EURTAC study [2] led to the approval of erlotinib (TARCEVA Genentech, Inc., South San Francisco, CA, USA and OSI Pharmaceuticals, Inc., Melville, NY, USA) in the same patient setting. In unselected populations, erlotinib has shown activity in about 10% of patients in terms of response rate and progression-free survival (PFS) [3,4,5]. For this reason, it has already been approved for the treatment of locally advanced or metastatic NSCLC after the failure of at least one prior chemotherapy regimen, regardless of *EGFR* status. However, recent work by Garassino and colleagues [6] showed that, in a second-line setting, chemotherapy is more effective than erlotinib in terms of response rate and progression-free survival (PFS) in *EGFR* wild type (wt) NSCLC patients. Two recent meta-analyses focusing on the role of TKIs in *EGFR* wt patients confirmed the superiority of chemotherapy over TKIs in terms of PFS but not of overall survival (OS) [7,8]. However, in each of the studies reviewed there was a subgroup of *EGFR* wt patients who obtained a clinical benefit from TKI treatment, suggesting that factors other than *EGFR* mutation may lead to TKI sensitivity in a small number of patients. Other biological mechanisms may, in fact, be responsible for TKI sensitivity in *EGFR* wild type NSCLC patients, such as *EGFR* expression or phosphorylation, *EGFR* amplification, *EGFR*-ligand expression or the involvement of other biomarkers that play a role in the *EGFR* pathway [9]. Moreover, highly sensitive methods for the evaluation of *EGFR* status can lead to the identification of activating mutations not highlighted by other conventional methodologies, justifying the response to TKIs [10].

In the present study we characterized NSCLC *EGFR* wt patients responding to erlotinib to identify potential biological markers of sensitivity and resistance to TKIs on the basis of their clinical features.

## 2. Results

In accordance with selection criteria, we identified 34 responsive patients among those treated with erlotinib in our institutions between January 2007 and June 2013. Median age was 69 years (range 44–88). Nineteen patients were male and 15 female. Twenty-five patients had adenocarcinoma (ADC), 6 had squamous cell carcinoma (SCC) and 3 had poorly differentiated carcinoma. Ten patients were current smokers, 8 former smokers and 8 non-smokers; smoking status was unknown for 8 patients. An equal number of non-responder patients, with similar characteristics for age, gender, smoking status and histotype, were analyzed. Patient characteristics are described in Table 1.

**Table 1:** Patient characteristics.

Characteristics	Total No.	Responders	Non-Responders
**Overall Case Series**	68	34	34
**Age, Years**			
≤65	27	13	14
>65	41	21	20
**Gender**			
M	38	19	19
F	30	15	15
**Smoking Habits**			
Smokers	25	10	15
Former	17	8	9
Never	12	8	4
NA	14	8	6
**Histology**			
ADC	52	25	27
SCC	11	6	5
Others	5	3	2
**Lines of Therapy**			
II	48	26	22
III	16	6	10
IV	4	2	2
**ECOG PS**			
0–1	58	32	26
2	6	1	5
n.a.	4	1	3

ADC, adenocarcinoma; SCC, squamous cell carcinoma; ECOG PS, Eastern Cooperative Oncology Group Performance Status; n.a., not available.

Overall, we found that 18 (53%) responsive and 16 (47%) non-responsive patients had a gene mutation highlighted by MassARRAY^®^ System. The different gene mutations found in the two groups are represented in Table 2. No significant correlations between mutations and clinical features were identified. However, in the responder group 10 patients with a mutation were former or never smokers (56%), 3 were smokers (17%) and 5 were unknown (28%). In the control arm, 5 patients were former or never smokers (31%), 8 were smokers (50%) and 3 were unknown (19%).

**Table 2:** Gene mutations identified using LungCarta kit.

Gene Mutation	Total No. (%)	No. of Responders (%)	No. of Non-Responders (%)
*EGFR* (sensitivity)	2 (3%)	2 (6%)	-
*EGFR* (resistance)	2 (3%)	-	2 (6%)
*KRAS*	10 (15%)	5 (15%)	5 (15%)
*PIK3CA*	3 (4%)	3 (9%)	-
*BRAF*	2 (3%)	2 (6%)	-
*p53*	6 (9%)	1 (3%)	5 (15%)
*STK11*	2 (3%)	2 (6%)	-
*NTRK2*	1 (1%)	1 (3%)	-
*NOTCH1*	4 (6%)	1 (3%)	3 ^§^ (8%)
*EPHA5*	2 (3%)	1 (3%)	1 (3%)
*AKT*	1 (1%)	-	1 (3%)
*MET*	1 (1%)	1 * (3%)	-

* This patient also had an *EGFR* L858R mutation; ^§^ One of these patients also had a *p53* G245C mutation.

Among responders, the analysis performed by MassARRAY^®^ System identified 2 patients with sensitizing *EGFR* mutations, one exon 19 deletion and one point mutation in exon 21 (L858R), previously missed by Pyrosequencing. The patient with the L858R mutation showed a concomitant mutation in *MET* (N375S). No *EGFR* sensitizing mutations were seen in non-responders, but 2 showed *EGFR* exon 20 mutations (P753S and L747S).

Mutation of *KRAS* gene was observed in 5 responders (15%) and in 5 non-responders (15%). In the former group, 2 patients had G12C mutation of *KRAS* (40%), one G12V (20%), one G12D (20%) and one G13D (20%). Among non-responders, 4 had G12C *KRAS* mutations (80%) and one G12V mutations (20%). All *KRAS* mutated tumors were adenocarcinoma (ADC).

Mutation of *p53* (R248Q) was identified in one responder with ADC, and in 5 non-responders (G245C, R273L, R249S, Y220C, R158C), 2 of these in a squamous cell carcinoma (SCC), 2 in ADC and one in large cell carcinoma. A higher *p53* mutation rate (15%) was observed in the non-responder patients as compared to responders (3%), (*p* = 0.09). All *p53* mutated non-responsive patients were smokers, whereas the *p53* mutated responsive patient had never been a smoker.

Mutation R2328W of the *NOTCH1* gene was present in one responsive SCC patient and in 3 non-responders (1 SCC and 2 ADC). Mutation S566Y of *EPHA5* was found in one responsive and one non-responsive patient, both with ADC. In the non-responder group, one patient with ADC showed an E17K mutation in the *AKT* gene. In the responsive group, 9 mutations (26%) were found in genes concerning proliferation pathways: *PIK3CA* (2 ADC with exon 9 E545K, and a poorly differentiated carcinoma with H1047R), *BRAF* (V600E in 2 ADC), *STK11* (V197fs 69 in one ADC and F354L in one SCC), *NTRK2* (L755L in a SCC) and *MET* (N375S). None of these mutations were identified in the control arm (*p* = 0.005).

No relevant correlations were found between different types of mutations and duration of response in the experimental group. Among mutated responsive patients, all but one had a stable disease and the median duration of response was 8 (range 6–10), 13.2 (range 7–22), 15 (range 9–20) and 11 (range 6–13) months for patients with mutations in *EGFR*, *KRAS*, *PIK3CA* and *BRAF*, respectively. One patient with a *PIK3CA* mutation showed partial response lasting for 20 months. Patients with mutation of *NTRK2*, *EPHA5*, *p53* and *NOTCH1* showed stable disease as a best response lasting for 6, 17, 19 and 10 months respectively (Table 3). Among responsive wt patients, 5 showed a partial response with a median duration of 10 months (range 7–12 months) while the others had stable disease as a best response with a median response duration of 9.5 months (range 6–14 months).

**Table 3:** Clinical-pathological characteristics and type of response in mutated responder patients.

Gene Mutations	Age	Gender	Smoking Habits	Histotype	Type and Duration of Response (Months)
***EGFR* sensitivity**					
Del 19	76	M	No	PDC	SD (6)
L858R *	77	F	No	ADC	SD (10)
***KRAS***					
G12V	64	M	Ex	ADC	SD (7)
G12C	71	M	Yes	ADC	SD (13)
G12C	75	M	n.a.	ADC	SD (15)
G12D	77	F	No	ADC	SD (9)
G13D	62	M	Yes	ADC	SD (22)
***PIK3CA***					
E545K	77	M	Ex	ADC	SD (9)
E545K	71	M	n.a.	ADC	SD (16)
H1047R	79	F	No	PDC	PR (20)
***BRAF***					
V600E	69	M	Yes	ADC	SD (16)
V600E	67	F	n.a.	ADC	SD (6)
***STK11***					
V197fs69	47	M	n.a.	ADC	SD (8)
F354L	75	M	Ex	SCC	SD (8)
***NTRK2***					
L755L	64	F	No	SCC	SD (6)
***EPHA5***					
S566Y	50	F	n.a.	ADC	SD (17)
*p53*					
R248Q	55	F	No	ADC	SD (19)
***NOTCH1***					
R2328W	66	M	Ex	SCC	SD (10)

* This patient also had a N375S *MET* mutation. PDC, poorly differentiated carcinoma; SD, stable disease; ADC, adenocarcinoma; SCC, squamous cell carcinoma; PR, partial response; n.a., not available; Ex, former smoker.

## 3. Discussion

EGFR-TKIs are a new class of drugs that have shown strong activity in NSCLC patients. Although the most significant results have been seen in *EGFR* mutated NSCLC, approximately 10% of *EGFR* wt patients may have an improvement in terms of response rate and progression-free survival [3,4,5]. At present, no clinical or biological parameters have been defined to identify patients with greater chances of response.

In our study, we performed a molecular characterization of *EGFR* wt NSCLC patients responsive to erlotinib in order to define markers predictive of response. *KRAS* mutation rate was equal in responders and non-responders (15% in the overall population of both groups, 20% in ADC). This result, even if obtained in a small number of cases, seems to confirm the data present in the literature according to which *KRAS* mutation is not predictive of non-response to TKIs. However, inconsistent data on the role of *KRAS* mutations in relation to TKI response are reported in the literature. Several authors found that *KRAS* mutated patients did not have a significant response benefit from TKI treatment, in different lines of therapy [11,12,13,14]. Conversely, several trials showed that no difference in terms of TKI response was identified based on *KRAS* status [15,16]. Other studies have analyzed the role of *KRAS* mutation subtype in relation to TKI response. A study by Metro *et al.* [17] showed that patients with *KRAS* codon 13 mutation showed a worse PFS and OS in comparison to patients with codon 12 *KRAS* mutations, although data were obtained in a small case series. Another study, conducted on a larger case series, showed that the G12C *KRAS* mutation is a negative predictor of efficacy of TKIs, in comparison to the other *KRAS* mutations [18]. According to the latter data, our case series showed that the G12C mutation was more frequently found in non-responders (80%) in comparison to responders (40%), suggesting that the G12C mutation may have a more specific value in predicting response to TKI.

In the responder group, 26% of mutations involved *PIK3CA* (9%), *BRAF* (6%), *STK11* (6%), *NTRK2* (3%) and *MET* (3%) genes were found, and these were not found in the non-responder group. All but one patient with these mutations had stable disease for a median time of approximately 10 months (range 6–16). One patient with a *PIK3CA* mutation showed a partial response for 20 months. Despite confirmation in several works that *PIK3CA*, *BRAF* and *MET* mutations can be major causes of acquired resistance to TKIs [19,20], limited data are available regarding their role at the baseline, prior to the start of any kind of treatment, and lack of information is probably due to their low incidence [21,22]. So far, given that all of the histological specimens used for analysis in our study have been collected at the baseline, our data suggest that these mutations may have a predictive role in patient selection that must be investigated in further prospective studies.

In the non-responder group, 29% of patients showed exon 20 *EGFR* mutations, *p53* or *NOTCH1* alterations, which are associated in the literature with TKI resistance [23,24,25]. In particular we found a percentage of *p53* gene mutations higher than that in responders (15% *vs.* 3%, respectively; *p* = 0.09) without a statistically significant value, probably due to the low number of cases. A recent study showed that non-disruptive mutations of the *p53* gene is an independent factor of shorter survival in advanced NSCLC with a possible predictive negative value of response to TKIs [23]. Moreover, a previous *in vitro* study demonstrated the central role of *p53* in the development of acquired resistance to EGFR inhibitors [24].

In the responder group, however, alterations of *p53* and *NOTCH1* were present in 5.9% of patients. The best response to erlotinib in these cases was stable disease with a median duration of 13.6 months (range 7–22 months). Prospective evaluations are needed to clarify whether the presence of these mutations before the start of cancer treatment could represent a potential criterion for patient selection. Moreover, taking into account the fact that we only evaluated hot spot *p53* mutations in our study, it might be interesting to extend our analysis to other gene alterations.

Finally, our data showed that by using a more sensitive method than Pyrosequencing, for example the MassARRAY^®^ System, it is possible to identify a higher number (6%) of *EGFR* mutated patients. These results are in line with a previous study, performed in an Asian case series, showing an increase of approximately 20% of *EGFR* mutation using Peptide Nucleic Acid (PNA) Clamping compared to Direct Sequencing [10], which has a lower sensitivity than the former. The higher increase in *EGFR* mutation found in this study is attributable to the fact that Direct Sequencing is a very low-sensitive methodology compared to PNA Clamping (10%–15% *vs.* 1% sensitivity, respectively), whereas we compared Pyrosequencing and MassARRAY^®^ System, which are more similar in terms of sensitivity (5%–8% *vs.* 2%–5%). Taken together, these data suggest that the use of a more sensitive sequencing technique should be recommended in order to also identify patients with a low level of *EGFR* mutation.

## 4. Experimental Section

### 4.1. Case Series

We conducted a retrospective evaluation of NSCLC *EGFR* wt patients treated between January 2007 and June 2013 at four institutions in the “Area Vasta Romagna”, a geographic area located in the North-East of Italy. *EGFR* status was determined at diagnosis using histological or cytological specimens and Pyrosequencing methodology, according to the clinical practice. All patients, in order to be eligible for selection, must have had histological specimens for analysis. Patients must have received at least one prior line of chemotherapy. Oral erlotinib was given at a dose of 150 mg/daily on a treatment cycle of 4 weeks. Treatment was continued until disease progression or unacceptable toxicity. Response evaluation was assessed according to Response Evaluation Criteria in Solid Tumor version 1.1 [26]. Patients with partial response or stable disease lasting for at least six months were defined as responsive. Patients with progressive disease or stable disease for less than five months were defined as non-responsive. Clinical data were collected from the databases of our institutions. Patients identified as responsive to erlotinib according to selection criteria were matched for age, gender, smoking status and histotype with an equal number of non-responder patients as a control arm. The study was approved by our Local Ethics Committee.

### 4.2. DNA Extraction

Both cytologic and histologic tumor specimens were accurately selected by qualified pathologists before DNA extraction. For cytologic smears, non-tumor cells were eliminated using a scalpel under an optical microscope while the remaining material, comprising almost 90% tumor cells, was scraped off the slides and placed in a test-tube. Cells were lysed in 50 mM KCl, 10 mM Tris–HCl pH 8.0, 2.5 mM MgCl_2_ and Tween-20 0.45%, with the addition of Proteinase K at a concentration of 1.25 mg/mL, overnight at 56 °C. Proteinase K was inactivated at 95 °C for 10 min after which samples were centrifuged twice to eliminate debris. Supernatants were assessed for DNA quality and quantity by Nanodrop (Celbio, Milan, Italy) and then underwent PCR amplification.

For paraffin-embedded samples, areas containing at least 70% of tumor cells were identified on haematoxylin-eosin-stained tissue sections, and 5-μM sections of the corresponding areas were macrodissected and collected in specific tubes. DNA extraction was performed as described for cytologic smears.

### 4.3. Molecular Determinations

A panel of 26 genes, for a total of 214 somatic mutations, were analyzed by MassARRAY^®^ System technology (Sequenom, San Diego, CA, USA), using the LungCarta Panel kit (Sequenom, San Diego, CA, USA). Analysis was performed using Typer 4.0 software (Sequenom, San Diego, CA, USA).

### 4.4. Statistical Analysis

Statistical analyses were performed using SPSS software (version 21.0, SPSS Inc, Chicago, IL, USA).

## 5. Conclusions

At present, no conclusion can be drawn in order to select patients sensitive to TKIs in second and further lines of treatment. The results of our study suggest that *p53*, *NOTCH1* and exon 20 *EGFR* mutations are apparently related to TKI resistance, whereas mutation of PIK3CA, BRAF, STK11, NTRK2 and MET, genes involved in proliferation pathways, were found in responders. *KRAS* mutations seem not to have a value predictive of response to TKI, although G12C alteration was found more frequently in non-responders. Finally, we confirmed that the use of highly sensitive methodologies could lead to the identification of under-represented *EGFR* mutations that could be associated with TKI sensitivity. These data need to be confirmed in a wider case series.
